# The importance of citizenship for deserving COVID-19 treatment

**DOI:** 10.1057/s41599-022-01311-4

**Published:** 2022-09-02

**Authors:** Marc Helbling, Rahsaan Maxwell, Simon Munzert, Richard Traunmüller

**Affiliations:** 1 Department of Sociology, University of Mannheim, Mannheim, Germany; 2 Department of Political Science, University of North Carolina at Chapel Hill, Chapel Hill, NC USA; 3 The Hertie School, Data Science and Public Policy, Berlin, Germany; 4 Department of Political Science, University of Mannheim, Mannheim, Germany

**Keywords:** Politics and international relations, Sociology

## Abstract

Immigrant non-citizens are often considered less deserving than citizens of welfare and other public services. The logic is that valuable and scarce public resources must be limited somehow, and the club of citizens is one way of drawing a boundary. In this paper, we examine how far that boundary extends, by analyzing the extent to which Germans prioritize citizens over non-citizens for access to life-saving healthcare. We implement a conjoint experiment to elicit preferences in the context of the COVID-19 pandemic. The data were collected between April 2020 and March 2021, in 23 waves of an online rolling cross-sectional survey with roughly 17,000 respondents. Our main finding is that citizens are viewed as more deserving of healthcare than non-citizen immigrants, a relationship that is sizeable and robust. Our findings have implications for debates about social boundaries and how to allocate resources in Western Europe.

## Introduction

Across Western Europe, there are intense political debates about who deserves access to public resources. One of the out-groups most often targeted as undeserving of scarce public resources is non-citizen immigrants (Magni, 2020). This dynamic of wanting to refuse social services to non-citizens is known as welfare chauvinism, and exists across countries and among numerous demographics within each country, with implications for elections, policy-making and the tone of public debates (Van Der Waal et al., 2013).

In this paper, we examine whether the desire to limit social services to citizens extends beyond welfare chauvinism to healthcare chauvinism. In particular, we examine whether the citizen/non-citizen boundary is salient for those who deserve access to life-saving COVID-19 treatment. Among the many challenges of COVID-19, one of the central public health problems is how to allocate healthcare resources. COVID-19 patients require intensive respiratory care treatment, which is a limited resource. When the number of COVID-19 patients exceeds the available resources, doctors have been forced to make difficult decisions; sometimes transferring patients to other regions or countries (Internationale, 2021), and sometimes sending people to die at home (Arshad, 2021).

We use original online survey data from Germany that ask for opinions about who is more deserving of life-saving healthcare. Respondents are presented with profiles of two patients, which vary on multiple dimensions, including whether they are German citizens or immigrants with a residence permit.

We find those non-citizen immigrants are seen as less worthy of life-saving COVID-19 treatment. Respondents are roughly 10 percentage points less likely to view non-citizen immigrants as worthy of COVID-19 treatment in comparison to citizens, which is a sizeable and robust relationship. The penalty is consistent among various subgroups of respondents, suggesting widespread healthcare chauvinism among German respondents. Moreover, the penalty remains even for non-citizen immigrants with characteristics that generally increase the likelihood of being considered deserving, such as being younger, having children, and not having a criminal record.

Our results have several implications and make several contributions. First, the strong evidence of healthcare chauvinism suggests the citizenship boundary is deep and meaningful in German society. This divide exists despite the fact that the immigrant-origin population is increasingly prominent and integrated into German popular culture and the labor market (Triadafilopoulos, 2019). This suggests immigrant integration could remain a long-term challenge in German society.

These findings are consistent with a growing number of studies that find respondents willing to discriminate against immigrants for access to COVID-19 treatment in Denmark (Larsen and Schaeffer, 2021) and Switzerland (Knotz et al., 2021). Our paper goes beyond those studies by implementing a research design that compares the citizenship divide to a broader range of additional patient characteristics. This allows us to explore the nuances of whether certain types of non-citizen immigrants are more likely to be seen as less deserving. In addition, our design includes 23 cross-sectional waves, which allows us to test whether public opinion on deservingness is sensitive to temporal fluctuations in the COVID-19 pandemic context.

This paper also contributes to a growing body of work on how the COVID-19 pandemic has revealed (and potentially exacerbated) existing societal divides. For example, other work suggests partisan divides structure people’s willingness to adopt public health measures in response to the threat of the COVID-19 pandemic (Gadarian et al., 2021; Greene et al., 2021). In addition, research finds that members of opposing political parties are seen as less deserving of critical COVID-19 treatment (Stoetzer et al., 2021). We build on this research by examining the importance of citizenship as an additional social boundary that shapes perceptions of deservingness.

Decisions about how to allocate scarce healthcare resources will always be difficult and involve challenging trade-offs. Our goal is not to judge the ideal standards for who is more deserving. Instead, we analyze how the citizenship boundary compares to other boundaries. In particular, we compare the citizenship boundary to the importance of utilitarian concerns about which patients will contribute more to society, which have become the standard benchmark for allocating scarce healthcare resources (Duch et al., 2021; Reeskens et al., 2021). This comparison allows us to place the citizenship boundary in context and provide perspective on its depth and salience.

Contrasting the citizenship boundary with utilitarian considerations allows us to engage in debates about ethical judgments and make a methodological contribution by using a salient real-world example. The standard approach to evaluating judgments about the value of life is the abstract ‘trolley problem’. This dilemma asks whether people would be willing to pull a lever to stop a runaway trolley from killing several people, and in the process divert it to a track where it killed a smaller number of people (Greene et al., 2001). There are many variations on this question, but they are all more abstract and unrealistic than our focus on the COVID-19 pandemic.

Admittedly, decisions about which lives deserve priority are typically reserved for specialized professionals (e.g. judges, police officers, doctors) (Parker and Mirzaali, 2020). However, the COVID-19 pandemic has heightened debates about the value of human life, which makes it useful for examining public opinion on which lives are more valuable (Hyland, 2020). Ordinary citizens will never need to make decisions about access to healthcare, but grounding our paper in meaningful and timely public debates provides sharp insight into how people evaluate whose lives are more worthy. These ethical judgments should have implications for social interactions and a wide range of public policy debates.

## Hypotheses

Our central research question is whether citizenship is a meaningful boundary for who is considered more deserving of life-saving healthcare. This inquiry is motivated by the fact that there is plenty of evidence that non-citizen immigrants face extensive discrimination in Europe, often justified by the logic that newly arrived outsiders are not sufficiently invested in the national community to warrant equal treatment (Koopmans et al., 2005). For example, there is evidence of bias against immigrants in the labor market (Dancygier and Laitin, 2014; Zschirnt and Ruedin, 2016), the housing market (Diehl et al., 2013; Quillian et al., 2020), as political candidates (Dancygier et al., 2021), in dealing with bureaucrats (Grohs et al., 2016; Hemker and Rink, 2017), and via stigmatization in everyday interactions (Essed, 1991; Tjaden et al., 2018).

It is plausible that widespread bias against non-citizen immigrants could lead to discriminatory behavior in access to healthcare. At the beginning of the COVID-19 pandemic, there were concerns in Germany that people with a migration background might be discriminated against in getting treatment in hospitals (Leubecher, 2020). Research in other contexts has shown that implicit biases against racial and ethnic minorities can exist among physicians (Stepanikova, 2012) and street-level bureaucrats (Andersen and Guul, 2019), especially during periods of intense stress (e.g. during a global pandemic). Moreover, COVID-19 originated in Asia and its global spread has been facilitated by travel. Under these conditions, people associated with other countries may appear threatening and harmful to society, making them less deserving of scarce resources (Roberto et al., 2020).

All of the above dynamics suggest that non-citizen immigrants may be an out-group that is seen as less deserving of life-saving healthcare, which generates our main hypothesis.

*H*_1_: *Non-citizen immigrants are less likely than citizens to be prioritized for access to COVID-19 treatment*.

An alternate hypothesis is that non-citizen immigrants and citizens are considered equally deserving of COVID-19 treatment. There is general public support—in Germany and across Western Europe—for broad access to healthcare. Everyone is vulnerable to illness and will get sick at some point, often for reasons beyond their control. As a result, deservingness is often broadly construed when debating access to healthcare (Jensen and Petersen, 2017). A stark contrast is unemployment benefits, where there are intense debates about the extent to which people can be held responsible for their unemployment (and thereby denied benefits) (van Oorschot, 2006). The fact that unemployment tends to be concentrated among low-status social groups further contributes to attempts to limit access to benefits. In comparison, healthcare is a much broader societal concern and therefore may have weaker boundaries for access.

Moreover, when healthcare treatment is rationed, priority is often based on utilitarian considerations about the value to society, and not ascriptive characteristics like national origin (Supady et al., 2021). The most prominent utilitarian consideration is the chance of survival after treatment: people with the greatest chance of survival are those who would be the best investment of scarce medical resources. This has been the key criterion in guidelines for treating COVID patients in Germany, according to the German Interdisciplinary Association for Intensive Care and Emergency Medicine (*Deutsche Interdisziplinäre Vereinigung für Intensiv-und Notfallmedizin*).

Admittedly, it is possible that short-term immigrants could be considered a less practical investment for the national community because they will soon leave. However, in this paper, we compare long-term residents who differ only in their origins and citizenship status. Therefore, to the extent that utilitarian considerations guide deservingness for COVID-19 treatment, there may be no difference between German citizens and non-citizen immigrants.

Finally, the rise of post-national and European Union-wide rights has limited the importance of nation–state citizenship as the basis for legitimate belonging to the community. Non-citizens in Europe can now access a wide range of social, political, and economic rights (Bloemraad, 2018; Soysal, 1994). In addition, immigrant political representation has steadily increased in recent decades across Europe, and these elected officials have been more aggressive at promoting rights for non-citizen immigrants (Ford and Jennings, 2020). There is also a core constituency in all European countries that supports open borders and more rights for newcomers.

Given all of the above considerations, access to healthcare—especially in the context of a global pandemic where everyone is vulnerable—can be considered a least likely case for detecting discrimination against non-citizen immigrants.

*H*_2_: *Non-citizen immigrants and citizens are equally likely to be prioritized for access to COVID-19 treatment*.

## Methods

### Sample

We fielded 23 survey waves between mid-April 2020 and March 2021. The first 13 waves (with roughly 500 respondents per wave) were fielded either weekly or biweekly between mid-April and mid-August 2020. Another 10 biweekly waves of *N* = 700 and *N* = 1000 were fielded between early November 2020 and March 2021.

Our data cover several phases of the COVID-19 pandemic in Germany. The early survey rounds cover the first peak of the pandemic in April 2020 as well as the relatively calmer summer of 2020. Our survey also covers the second big wave of the pandemic in fall 2020 and winter 2020/21, as well as the beginning of the third wave in March 2021. By collecting data across all of these time periods, we can explore whether preference structures varied according to different phases of the pandemic.

The survey was conducted online by the firm Respondi.^1^ Respondents were 18 years or older and the sample was designed to be nationally representative according to gender, age, and education. Roughly 20 percent of the respondents were either born abroad or had at least one parent who was born abroad.^2^ Descriptive statistics are in Appendix Table 1.

### Case selection

The German case has many similarities to other West European countries. For example, non-citizen immigrants face discrimination in many areas of life in Germany, as they do elsewhere in Western Europe (Goodman, 2014). At the same time, the welfare state is fairly well-developed in Germany (and elsewhere in Western Europe), providing access to strong public services (including healthcare), many of which are available to citizens and non-citizens (Soysal, 1994; Van Der Waal et al., 2013). As a result, Germany is similar to many other Western European countries as a place where non-citizen immigrants are vulnerable to stigmatization but can also expect to access robust public services like healthcare.

For much of the 20th century, it was very difficult to acquire German citizenship, which meant the citizen/non-citizen divide was a bright line that marked more social exclusion than elsewhere in Western Europe Joppke (1999). However, in recent years, German citizenship law has liberalized and converged towards European norms (Koopmans et al., 2012; Schmid, 2021). While every country has unique particularities, the citizen/non-citizen divide in Germany is now comparable to the citizen/non-citizen divide across Western Europe (Geddes and Scholten, 2016). In short, the basic structure of German society as it pertains to our analysis should be similar to societies across Western Europe.

### Experimental design

We conducted a preregistered paired between-subject conjoint with forced choice experiment.^3^ Each respondent was presented with two hypothetical patients and asked to choose the patient more deserving of treatment for COVID-19. The hypothetical patients were described with profiles that include seven attributes: chances of survival, age, occupation, having school-aged children, criminal record, gender, and citizenship status. The specifics of each attribute were randomized across respondents.

Our primary research question is whether citizenship status affects respondents’ answers. We do not distinguish between German citizens with immigrant and native origins. Given the exclusive nature of German identity, we do not expect many respondents to associate immigrants with German citizens. To the extent that some respondents do imagine immigrant-origin German citizens, that should reduce the likelihood of discrimination against non-citizen immigrants. This suggests our results are a conservative estimate of bias against non-citizen immigrants.

We included the other attributes to present a well-rounded profile of the hypothetical patients and to benchmark any effects of citizenship status against utilitarian considerations that could be considered more ‘legitimate’ bases for maximizing societal benefits (Supady et al., 2021). For example, chances of survival are a direct calculation of the odds that investing in healthcare will lead to a successful outcome. Age is a practical consideration because younger patients have more of their (working) lives ahead of them, and could be considered a better investment for societal resources.

We present four occupations (cooks, nurses, professors, and doctors), which vary on two dimensions: social status and system relevance. If social status drives the calculation for who is more worthy, then professors and doctors should be chosen. If system relevance drives the calculation for who can provide more societal value during a pandemic, then nurses and doctors should be chosen. Having school-aged children is a responsibility that is an investment in the future of society, and could be considered more valuable for the broader community. Finally, having a criminal record could be considered evidence that the patient detracts from society. We include gender to present a fuller picture of the patient, but we have no expectations for whether men or women should be seen as more deserving of healthcare.

Each respondent was exposed to one pair of patients. The order of the profile characteristics was fixed across all respondents.^4^ Respondents were presented with the following text and then asked to choose between the two patients. Table 1 presents the full set of category options that could have been presented to respondents.

**Table 1:** Patient attributes.

Attribute	Randomized categories
Gender	Male, Female
Age	30 years old, 44 years old, 64 years old
Chance of survival in ICU	20%, 50%, 80%
Occupation	Medical doctor, professor, nurse, cook
Children	Has school-aged children, has no children
Migration status	German citizen, migrant with residence permit
Criminal record	Has criminal record, has no criminal record

Experts believe that the German health care system is well prepared to help all persons with severe coronavirus symptoms. Due to the strong increase of cases in critical conditions, it could however soon become necessary to decide who is admitted first to the intensive care unit and be put on a ventilator. According to you, which of the following two patients should be admitted first to the intensive care unit? Please consider that the indicated chances of survival already account for factors like age and gender. For example, a 30-year-old patient with 40% chances of survival has the same chances of survival as an 80 years old patient with 40% chances of survival, namely 40%.

### Statistical analysis

We estimate *average marginal component effects* (AMCEs) and *average component interaction effects* (ACIE) for the profile attributes. Those effect estimates are equivalent to OLS estimates. Standard errors were clustered on survey waves. All tests reported are two-tailed. While our conjoint experiment includes multiple profile dimensions and attributes, we did not correct the *p*-values for multiple hypotheses. All analyses are based on unweighted data and simple list-wise deletion of missing values.

## Results

### Main effects on Triage decisions

**Fig. 1:**
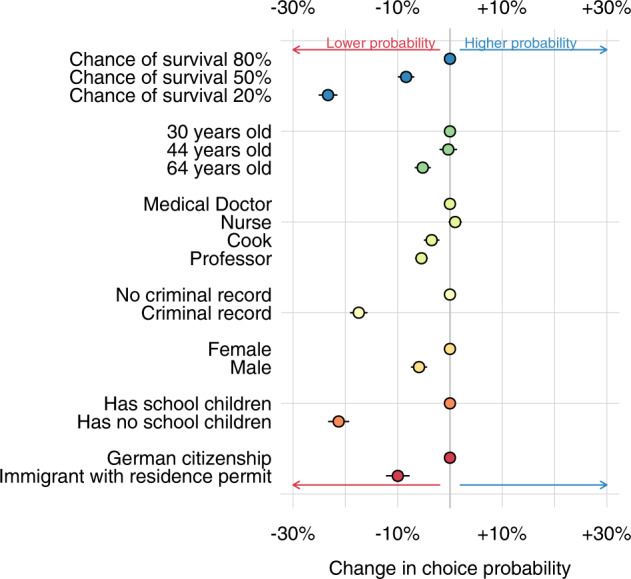
For each patient attribute, the reference category is listed first and represented at 0 on the *x*-axis (80% chance of survival, 30 years old, Medical Doctor, No criminal record, Female, Has school-aged children, German citizen). For the other categories, dots (with 95% confidence intervals) are the difference in the average marginal component effects (AMCE) between the reference category and the category listed on the *y*-axis. Pooled survey waves 1–23.

Figure 1 presents our main results. Recall that each of the seven patient attributes in our conjoint has multiple categories, which were randomly presented to each respondent. To place the ACMEs in context and facilitate a clear interpretation of their relative effects, we select a reference category for each attribute, and Fig. 1 presents the difference in choice probability between the reference category and the other response options.

In line with utilitarian considerations, the chance of survival is the largest predictor of who is more deserving of care. Patients with a survival chance of 20% are 26 percentage points less likely than patients with a survival chance of 20% to be selected as a priority for care (those with a survival chance of 50% are 15 percentage points less likely to be selected). The second and third largest effects are also in line with utilitarian considerations. Not having school-age children decreases the likelihood of being selected by 21 percentage points relative to patients with school-age children. Patients with a criminal record are 18 percentage points less likely than those without a criminal record to be given priority for treatment.

The next largest penalty is for non-citizen immigrants, who are 10 percentage points less likely than citizens to be chosen for treatment. This penalty is larger than the penalties for specific occupations, ages, or for male as opposed to female patients. Decisions about the limited access to life-saving care will always involve difficult trade-offs, but it is especially striking that the citizenship difference is more salient than the difference across occupations and ages.

All occupations offer value to society, but in the context of a global pandemic, one might imagine that system-relevant occupations would be highly-prioritized. Figure 1 suggests system relevance does play a role in who is seen as more worthy of treatment, as nurses (higher in system relevance, lower in social status) are more likely to be prioritized than professors (higher in social status, lower in system relevance). However, these differences are smaller than the citizen/non-citizen gap. Similarly, while people of all ages offer value to society, in the context of a pandemic one might imagine that younger people would be highly prioritized as an investment in developing a new future for society. Figure 1 suggests age does play a role in deservingness, as 64-year-olds are less likely to be prioritized than younger categories. However, for German respondents, the difference between citizens and non-citizens is more important.

### Effect changes over time

We collected our data in 23 waves over 12 months, as the COVID-19 pandemic went through many twists and turns, so it is possible that respondents’ evaluations varied according to specific temporal contexts. Most notably, during periods in which positive COVID-19 cases were rising, respondents may have experienced more generalized fear and anxiety. Research suggests that fear and anxiety are powerful emotions that trigger hostility towards out-groups like immigrants and ethnic minorities (Albertson and Gadarian, 2015; Banks, 2014), so it is possible that bias against non-citizen immigrant patients was concentrated during periods when COVID-19 felt more threatening.

To explore whether our results are consistent across the time period, we plot wave-specific AMCEs and their 95% confidence intervals in a time series plot (see Appendix Fig. 1). For the most part, we find that the effects of patient characteristics on the probability of deserving treatment are consistent across the 23 waves. Most importantly, non-citizen immigrants are considered less worthy of treatment than German citizens across all waves. Nonetheless, the size of the penalty for non-citizen immigrants does change over time: it is smaller in waves 14–23 than in waves 1–13 (this difference is statistically significant at the 95% level).

One possible explanation for this shift could be that we adjusted the research design in waves 14–23 to test for different reactions to EU non-citizen immigrants as opposed to non-EU non-citizen immigrants (described in detail below). However, our results (described below) find no difference in reactions to EU and non-EU non-citizen immigrants. Therefore, it is unlikely that the smaller penalty in waves 14–23 is a result of being presented with more specific types of non-citizen immigrants. Another possibility is that the composition of respondents shifted in the latter waves. However, Appendix Table 1 presents descriptive statistics and indicates similar respondent profiles in waves 1–13 and waves 14–23.

One could imagine that the size of the bias against non-citizen immigrants rises during periods of heightened concern about COVID-19 and declines when COVID-19 is less threatening. However, COVID-19 fluctuations do not match the shifts in bias against non-citizen immigrants in our results. The reduction in the penalty for non-citizen immigrants occurred during waves 14–23, which was the timing of the second big wave of COVID-19 (winter 2020/21), when anxiety may have been higher than in summer 2020 (when the penalty was larger). Alternate research designs are necessary to more thoroughly investigate how (pandemic-related) anxiety may shape changing responses to non-citizen immigrants. Nonetheless, the key finding of this paper is that non-citizen immigrant are consistently seen as less deserving of COVID-19 treatment than German citizens.

### Variation across respondents

Research on bias tends to find variation in the likelihood of discrimination across different subgroups. This is relevant for our analyses because some Germans are more likely than others to be biased against immigrants. If the discrimination against non-citizen immigrant patients only exists among Germans who hold more negative attitudes against immigrants in general, that would suggest healthcare chauvinism is a limited phenomenon. If the penalty exists across all German respondents, that would suggest healthcare chauvinism is a widespread phenomenon.

**Fig. 2:**
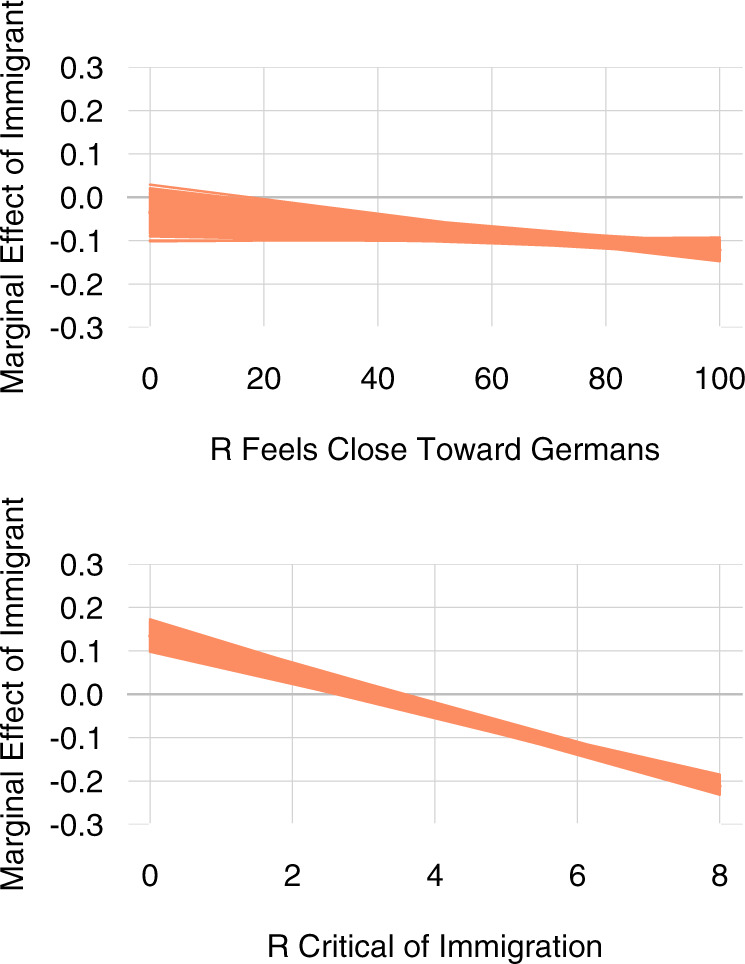
Variation in the non-citizen immigrant penalty across respondents (marginal effects and simulated 95% confidence intervals). Pooled survey waves 1–23.

Figure 2 plots the marginal effect of being a non-citizen immigrant on the probability of being selected for priority treatment across two types of respondent variation. The top panel plots result according to the extent to which respondents feel close to Germans, a measure of national identity attachment that could signal bias against outsiders.^5^ Here the results indicate that respondents at the extreme low end of the closeness to Germans scale do not distinguish between citizens and immigrant non-citizens for healthcare access. As respondents feel closer to Germans, there is a larger penalty (statistically significant at the 95% level) against non-citizen immigrants. Figure 2 suggests variation in the intensity of bias, but it also suggests that most Germans exhibit some bias against non-citizen immigrant patients. The only respondents without non-citizen bias are those on the extreme end of feeling distant from Germans.

The bottom panel of Fig. 2 plots the marginal effect of a patient being a non-citizen immigrant according to whether respondents are critical of immigration.^6^ Here we see that respondents who support immigration (on the far left of the scale) are actually biased in favor of non-citizen immigrant patients. As respondents become more critical of immigration, that bias moves against the non-citizen immigrant patient. In short, both panels of Fig. 2 suggest variation in the non-citizen penalty across different types of respondents. Nonetheless, in both panels, the bias against non-citizens is widespread and statistically significant (at the 95% level) across most of the closeness to Germans/critical of immigration scales.

Although healthcare chauvinism may be widespread among German residents, it is not universal. Additional analyses suggest that the penalty against non-citizen immigrants does not exist among respondents who are not German citizens (full results are in Appendix Fig. 2). This distinction is intuitive. Respondents without German citizenship are more likely to identify with the non-citizen patient, which should decrease their incentive to discriminate against that type of patient.^7^

### Variation among patients

‘Non-citizen immigrant’ is a broad category, encompassing people with different demographic characteristics and different levels of utility for society. It is possible that German respondents view non-citizen immigrants in general as less worthy of life-saving healthcare, but are willing to view non-citizen immigrants as deserving if they have certain characteristics. For example, Fig. 1 suggests that patients with high survival chances, school-aged children, and no criminal record are most likely to be viewed as deserving, so perhaps non-citizen immigrants with those characteristics are viewed no differently than citizens with those characteristics.

**Fig. 3:**
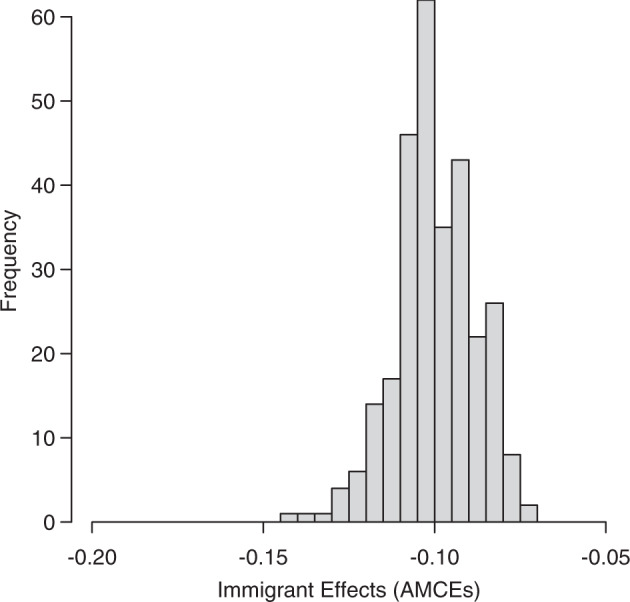
Distribution of Immigrant Effects (AMCEs) across *J* = 288 unique profile combinations. Pooled survey waves 1–23.

Figure 3 explores this possibility by plotting the distribution of the non-citizen effect across all 288 unique profile combinations in our experiment. Across all of these combinations, non-citizens are always penalized relative to citizens, with effect sizes ranging from seven to fourteen percentage points. In other words, non-citizen immigrants are still considered less of a priority for COVID-19 treatment, even when they have all the characteristics that otherwise lead to large increases in priority.

**Fig. 4:**
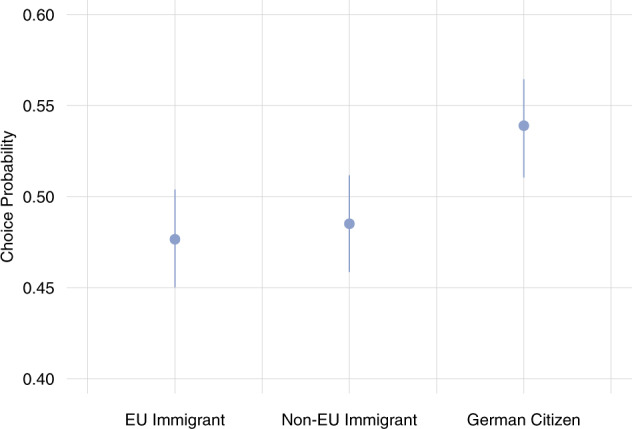
Comparison across immigrant status—EU migrants, non-EU migrants, German citizens (choice probability and simulated 95% confidence intervals). Pooled survey waves 14–23.

Another possibility is that deservingness varies according to the immigrants’ origins. In Germany, European-origin immigrants tend to be less stigmatized than non-European-origin immigrants (Partridge, 2012). To test whether this distinction affects being seen as worthy of healthcare, we added an additional category in waves 14–23 to distinguish between EU immigrants, non-EU immigrants, and German citizens. Figure 4 presents results from this comparison and shows that both EU and non-EU immigrants are penalized to the same extent. This suggests that priorities for access to life-saving COVID-19 healthcare are driven by concern about the national boundary and are not necessarily confounded with additional racial or ethnic considerations.^8^

## Discussion and conclusion

The COVID-19 pandemic has raised difficult ethical questions about whose life is more valuable. These questions have been present throughout the pandemic in debates about which parts of society to prioritize when implementing lockdowns and other public health measures. We use that salient and timely pandemic context of who should be prioritized to explore German opinions about who deserves priority access to life-saving COVID-19 healthcare. Our results suggest a strong public preference for giving priority to German citizens as opposed to non-citizen immigrants. This preference is consistent across a wide range of German respondents and regardless of non-citizen immigrant patients’ other characteristics.

Our results indicate that German respondents follow the guidelines of medical associations by giving the highest priority to patients with the highest chances of survival. This is evidence that utilitarian concerns matter, as one might expect for difficult ethical decisions about who deserves access to life-saving care. However, the relatively-large penalty for non-citizens is evidence that national identity and citizenship boundaries matter as well. Our contribution is in highlighting how far that priority extends, namely to the area of life-saving healthcare treatment, where ethical considerations suggest citizenship boundaries should matter less.

Given the moral imperative to treat the sick, it is reasonable to consider access to life-saving healthcare a least likely case for discrimination against non-citizen immigrants. One interpretation of our findings is that the boundary with immigrant non-citizens in Germany is deep and stark and will remain important. To the extent that is true, non-citizen immigrants are likely to face stigmatization and discrimination in multiple areas of life. Moreover, this bias might extend to support for public policies that limit the right of non-citizen immigrants to access various public goods (including healthcare). Research should continue to explore these issues and track how it develops in the future.

Future research should also go further to explore the contours of this healthcare chauvinism and how it might vary for different types of healthcare services and different types of immigrants. It would also be useful to test the extent of healthcare chauvinism in a range of country cases, where public health services are both more and less extensive than in Germany. The literature on welfare chauvinism and healthcare chauvinism is based on assumptions about strong public services that are funded through taxation and available for deserving members of society. Countries outside of Western Europe, with greater reliance on private goods, may have different calculations about the deservingness of non-citizen immigrants.

## Supplementary information

Appendix

## Data Availability

The data used in this article and replication code are available on the Harvard Dataverse: 10.7910/DVN/3KBY7Y. Traunmueller, Richard, 2022, “Replication Data for: Maxwell, Helbling, Munzert & Traunmueller: The importance of citizenship for deserving COVID-19 treatment”, 10.7910/DVN/3KBY7Y, Harvard Dataverse, V1.

## Footnotes

1 For more information on the Respondi access panel: https://www.respondi.com/.

2 The study design was approved by the Ethics Commission of the Social Science Research Center Berlin (WZB)—study 2020-0-88—and the data collection complies with the General Data Protection Regulation (GDPR) of the European Union and the Declaration of Helsinki. Informed consent was obtained from all participants.

3 The study was pre-registered on 21 April 2020 when the first wave was in the field. The pre-registration plan can be accessed here: https://osf.io/ga8r9.

4 The randomization of attributes was programmed differently across different waves. In the first 14 waves, the two patients could never have the same characteristics on any of the attributes, in order to avoid the possibility that participants were asked to choose between two identical patients. In waves 15–23, the experiment was the same for 40% of the sample. For the other 60% of the sample, the attributes were completely randomized. As seen in Appendix Fig. 3, the programming did not affect our findings.

5 People were asked the following question on a scale from 0 (not at all connected) to 10 (very much connected): “How strongly do you feel connected to the following groups?” Among several items, here we use their response to ‘People in Germany’.

6 People were asked to indicate on a scale from 0 (don’t agree at all) to 7 (fully agree): “How much do you agree with the following statement? Germany should restrict immigration.”

7 Our pre-registration plan outlined several more interactions between patient and respondent characteristics, which we present in Appendix Fig. 4 and Appendix Tables 3–15. None of these directly relate to the question of discrimination against non-citizen immigrant patients.

8 This may seem surprising, given the conventional wisdom that attitudes vary towards migrants from different countries. One explanation is that more specific out-group categories (e.g. ‘Muslims’ or ‘Syrians’) would have elicited more extreme discrimination. Another possibility is that healthcare chauvinism is primarily a citizenship divide. Future research should explore this in more detail.
