# Supplementary material for: The importance of citizenship for deserving COVID-19 treatment
Source: Humanit Soc Sci Commun. 2022 Sep 2;9(1):302. doi: 10.1057/s41599-022-01311-4 (PMC9436734; doi:10.1057/s41599-022-01311-4)
Supplement: Supplementary file 1 — Appendix [file 41599_2022_1311_MOESM1_ESM.pdf]

The importance of citizenship for deserving COVID-19 treatment
Appendix

Marc Helbling* Rahsaan Maxwell† Simon Munzert‡
Richard Traunmüller§

*University of Mannheim, Sociology
†University of North Carolina at Chapel Hill, Political Science
‡The Hertie School, Data Science and Public Policy
§University of Mannheim, Political Science

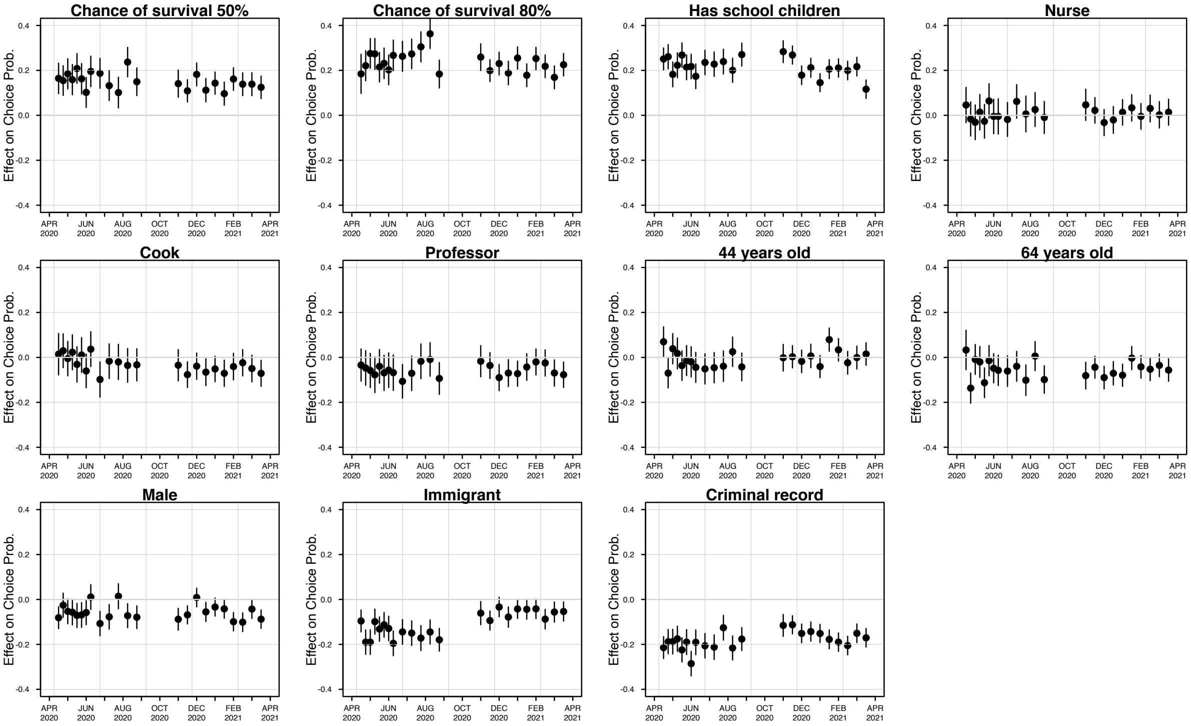

Figure 1: Wave-specific effects (ACMEs). Pooled survey waves 1-23.

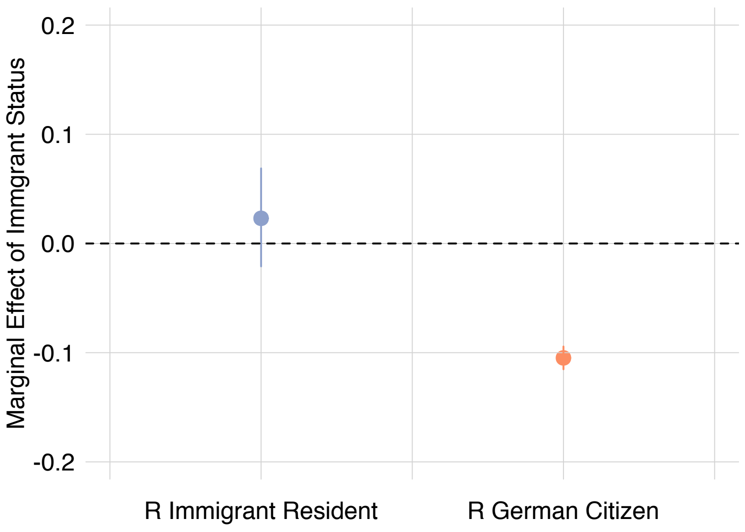

Figure 2: Interactions between patient and respondent immigrant status (Average Component Interaction Effects (ACIEs) and 95% confidence intervals). Pooled survey waves 1-23.

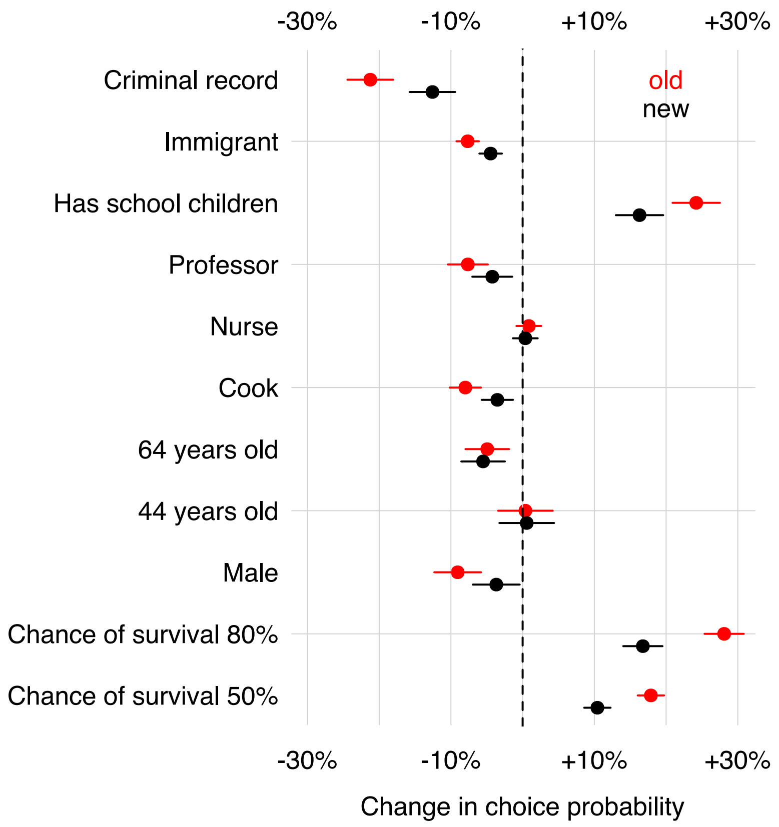

Figure 3: Comparison of old vs. new programming versions. Pooled survey waves 15-23.

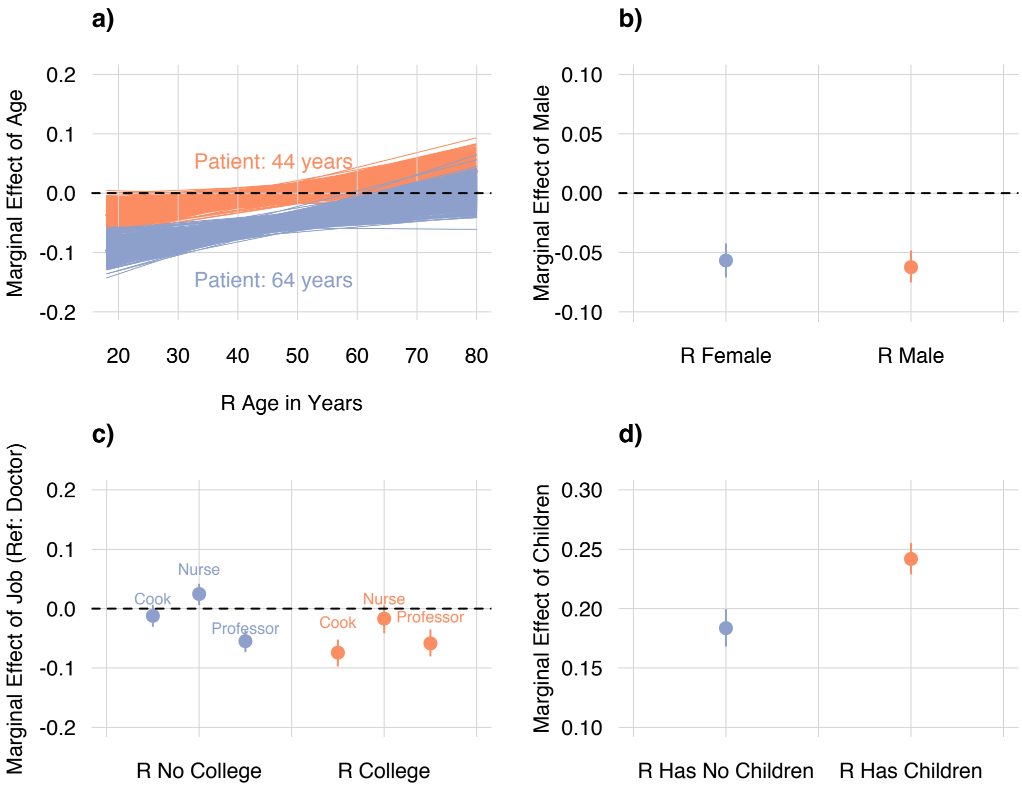

Figure 4: Interactions between patient and respondent characteristics (Average Component Interaction Effects (ACIEs) and 95% confidence intervals). Pooled survey waves 1-23.

Tables

Table 1: Descriptive statistics

	All Waves			
	Mean	SD	Min.	Max.
Female	0.50	0.50	0.00	1.00
Age	45.07	14.55	18.00	99.00
Education (Abitur)	0.37	0.48	0.00	1.00
German	0.95	0.21	0.00	1.00
Has children	0.54	0.50	0.00	1.00
Left-Right-Ideology	5.71	1.95	0.00	11.00
Immigration Attitudes	5.36	2.37	0.00	8.00

	Waves 1-13			
	Mean	SD	Min.	Max.
Female	0.50	0.50	0.00	1.00
Age	45.32	14.73	18.00	99.00
Education (Abitur)	0.38	0.48	0.00	1.00
German	0.95	0.22	0.00	1.00
Has children	0.52	0.50	0.00	1.00
Left-Right-Ideology	5.74	1.94	0.00	11.00
Immigration Attitudes	5.38	2.37	0.00	8.00

	Waves 14-23			
	Mean	SD	Min.	Max.
Female	0.50	0.50	0.00	1.00
Age	44.81	14.34	18.00	99.00
Education (Abitur)	0.36	0.48	0.00	1.00
German	0.96	0.20	0.00	1.00
Has children	0.55	0.50	0.00	1.00
Left-Right-Ideology	5.69	1.95	0.00	11.00
Immigration Attitudes	5.35	2.37	0.00	8.00

Table 2: Table of main results from the triage experiment

	Estimate	Std. Error	t value	Pr(>|t|)
(Intercept)	0.47	0.01	44.02	0.00
Chance of survival 50%	0.15	0.01	20.69	0.00
Chance of survival 80%	0.23	0.01	27.18	0.00
Male	-0.06	0.01	-8.14	0.00
44 years old	-0.00	0.01	-0.39	0.69
64 years old	-0.05	0.01	-7.16	0.00
Cook	-0.03	0.01	-4.94	0.00
Nurse	0.01	0.01	1.70	0.09
Professor	-0.05	0.01	-9.72	0.00
Has school children	0.21	0.01	21.91	0.00
Immigrant	-0.10	0.01	-9.08	0.00
Criminal record	-0.17	0.01	-21.97	0.00

Table 3: Table of interaction results: Respondent vs. patient age

	Estimate	Std. Error	t value	Pr(>|t|)
(Intercept)	0.53	0.02	26.78	0.00
Chance of survival 50%	0.15	0.01	21.21	0.00
Chance of survival 80%	0.23	0.01	27.98	0.00
Male	-0.06	0.01	-8.52	0.00
44 years old	-0.06	0.03	-2.09	0.04
64 years old	-0.13	0.02	-6.83	0.00
Cook	-0.04	0.01	-5.16	0.00
Nurse	0.01	0.01	1.70	0.09
Professor	-0.06	0.01	-10.36	0.00
Has school children	0.21	0.01	22.82	0.00
Immigrant	-0.10	0.01	-9.60	0.00
Criminal record	-0.17	0.01	-22.77	0.00
R: Age	-0.00	0.00	-3.07	0.00
44 years old * R: Age	0.00	0.00	1.90	0.06
64 years old * R: Age	0.00	0.00	3.79	0.00

Table 4: Table of interaction results: Respondent vs. patient gender

	Estimate	Std. Error	t value	Pr(>|t|)
(Intercept)	0.48	0.01	39.82	0.00
Chance of survival 50%	0.15	0.01	21.35	0.00
Chance of survival 80%	0.23	0.01	28.15	0.00
Male	-0.06	0.01	-4.28	0.00
44 years old	-0.00	0.01	-0.58	0.56
64 years old	-0.05	0.01	-7.65	0.00
Cook	-0.04	0.01	-5.16	0.00
Nurse	0.01	0.01	1.65	0.10
Professor	-0.06	0.01	-10.34	0.00
Has school children	0.21	0.01	22.77	0.00
Immigrant	-0.10	0.01	-9.60	0.00
Criminal record	-0.17	0.01	-22.75	0.00
R: Female	0.00	0.01	0.22	0.83
Male * R: Female	-0.01	0.02	-0.33	0.74

Table 5: Table of interaction results: Respondent vs. patient SES

	Estimate	Std. Error	t value	Pr(>|t|)
(Intercept)	0.48	0.01	43.46	0.00
Chance of survival 50%	0.15	0.01	21.30	0.00
Chance of survival 80%	0.23	0.01	27.89	0.00
Male	-0.06	0.01	-8.53	0.00
44 years old	-0.00	0.01	-0.57	0.57
64 years old	-0.05	0.01	-7.62	0.00
Cook	-0.01	0.01	-1.14	0.25
Nurse	0.02	0.01	2.63	0.01
Professor	-0.05	0.01	-8.23	0.00
Has school children	0.21	0.01	22.86	0.00
Immigrant	-0.10	0.01	-9.67	0.00
Criminal record	-0.17	0.01	-22.77	0.00
R: High education	0.03	0.01	2.38	0.02
Cook * R: High education	-0.06	0.02	-3.06	0.00
Nurse * R: High education	-0.04	0.02	-2.29	0.02
Professor * R: High education	-0.00	0.01	-0.29	0.77

Table 6: Table of interaction results: Respondent vs. patient has children

	Estimate	Std. Error	t value	Pr(>|t|)
(Intercept)	0.50	0.01	43.78	0.00
Chance of survival 50%	0.15	0.01	21.33	0.00
Chance of survival 80%	0.23	0.01	28.20	0.00
Male	-0.06	0.01	-8.45	0.00
44 years old	-0.00	0.01	-0.58	0.56
64 years old	-0.05	0.01	-7.68	0.00
Cook	-0.03	0.01	-5.07	0.00
Nurse	0.01	0.01	1.64	0.10
Professor	-0.06	0.01	-10.43	0.00
Has school children	0.18	0.01	14.02	0.00
Immigrant	-0.10	0.01	-9.60	0.00
Criminal record	-0.17	0.01	-22.87	0.00
R: Has children	-0.03	0.01	-3.96	0.00
Has school children * R: Has children	0.06	0.01	4.44	0.00

Table 7: Table of interaction results: Respondent vs. patient immigrant status

	Estimate	Std. Error	t value	Pr(>|t|)
(Intercept)	0.41	0.02	21.27	0.00
Chance of survival 50%	0.15	0.01	21.07	0.00
Chance of survival 80%	0.23	0.01	28.17	0.00
Male	-0.06	0.01	-8.46	0.00
44 years old	-0.00	0.01	-0.59	0.56
64 years old	-0.05	0.01	-7.67	0.00
Cook	-0.04	0.01	-5.16	0.00
Nurse	0.01	0.01	1.62	0.11
Professor	-0.06	0.01	-10.30	0.00
Has school children	0.21	0.01	22.79	0.00
Immigrant	0.02	0.03	0.81	0.42
Criminal record	-0.17	0.01	-22.86	0.00
R: German	0.08	0.02	4.10	0.00
Immigrant * R: German	-0.13	0.03	-3.68	0.00

Table 8: Table of interaction results: Respondent nationalist attitude vs. patient immigrant status

	Estimate	Std. Error	t value	Pr(>|t|)
(Intercept)	0.45	0.02	27.44	0.00
Chance of survival 50%	0.15	0.01	21.61	0.00
Chance of survival 80%	0.23	0.01	28.62	0.00
Male	-0.06	0.01	-8.46	0.00
44 years old	-0.00	0.01	-0.49	0.62
64 years old	-0.05	0.01	-7.52	0.00
Cook	-0.04	0.01	-5.29	0.00
Nurse	0.01	0.01	1.60	0.11
Professor	-0.06	0.01	-10.13	0.00
Has school children	0.22	0.01	23.05	0.00
Immigrant	-0.04	0.02	-1.59	0.11
Criminal record	-0.18	0.01	-22.68	0.00
R: Nationalism	0.00	0.00	3.32	0.00
Immigrant * R: Nationalism	-0.00	0.00	-3.24	0.00

Table 9: Table of interaction results: Respondent immigration attitude vs. patient immigrant status

	Estimate	Std. Error	t value	Pr(>|t|)
(Intercept)	0.35	0.01	24.72	0.00
Chance of survival 50%	0.15	0.01	21.45	0.00
Chance of survival 80%	0.23	0.01	27.19	0.00
Male	-0.06	0.01	-8.42	0.00
44 years old	-0.00	0.01	-0.54	0.59
64 years old	-0.05	0.01	-7.62	0.00
Cook	-0.04	0.01	-5.62	0.00
Nurse	0.01	0.01	1.59	0.11
Professor	-0.06	0.01	-10.83	0.00
Has school children	0.21	0.01	22.65	0.00
Immigrant	0.13	0.01	9.32	0.00
Criminal record	-0.17	0.01	-22.68	0.00
R: Immigration Attitude	0.03	0.00	15.72	0.00
Immigrant * R: Immigration Attitude	-0.04	0.00	-11.93	0.00

Table 10: Table of interaction results: Respondent left-right ideology vs. patient occupation

	Estimate	Std. Error	t value	Pr(>|t|)
(Intercept)	0.45	0.02	19.76	0.00
Chance of survival 50%	0.15	0.01	21.33	0.00
Chance of survival 80%	0.23	0.01	28.05	0.00
Male	-0.06	0.01	-8.42	0.00
44 years old	-0.00	0.01	-0.55	0.58
64 years old	-0.05	0.01	-7.65	0.00
Cook	0.03	0.03	0.91	0.36
Nurse	0.05	0.02	2.34	0.02
Professor	-0.04	0.03	-1.16	0.25
Has school children	0.21	0.01	22.72	0.00
Immigrant	-0.10	0.01	-9.56	0.00
Criminal record	-0.17	0.01	-22.83	0.00
R: Left-right ideology	0.01	0.00	1.75	0.08
Cook * R: Left-right ideology	-0.01	0.00	-2.35	0.02
Nurse * R: Left-right ideology	-0.01	0.00	-1.96	0.05
Professor * R: Left-right ideology	-0.00	0.01	-0.59	0.55

Table 11: Table of interaction results: Respondent left-right ideology vs. patient age

	Estimate	Std. Error	t value	Pr(>|t|)
(Intercept)	0.46	0.02	28.27	0.00
Chance of survival 50%	0.15	0.01	21.39	0.00
Chance of survival 80%	0.23	0.01	28.06	0.00
Male	-0.06	0.01	-8.45	0.00
44 years old	0.02	0.02	0.82	0.41
64 years old	-0.01	0.02	-0.68	0.50
Left-right ideology	0.00	0.00	1.69	0.09
Cook	-0.03	0.01	-5.08	0.00
Nurse	0.01	0.01	1.66	0.10
Professor	-0.06	0.01	-10.34	0.00
Has school children	0.21	0.01	22.75	0.00
Immigrant	-0.10	0.01	-9.61	0.00
Criminal record	-0.17	0.01	-22.80	0.00
44 years old * R: Left-right ideology	-0.00	0.00	-1.00	0.32
64 years old * R: Left-right ideology	-0.01	0.00	-1.87	0.06

Table 12: Table of interaction results: Respondent left-right ideology vs. patient has children

	Estimate	Std. Error	t value	Pr(>|t|)
(Intercept)	0.46	0.01	35.26	0.00
Chance of survival 50%	0.15	0.01	21.46	0.00
Chance of survival 80%	0.23	0.01	28.18	0.00
Male	-0.06	0.01	-8.53	0.00
44 years old	-0.00	0.01	-0.58	0.56
64 years old	-0.05	0.01	-7.59	0.00
Cook	-0.04	0.01	-5.14	0.00
Nurse	0.01	0.01	1.61	0.11
Professor	-0.06	0.01	-10.33	0.00
Has school children	0.27	0.02	10.92	0.00
R: Left-right ideology	0.00	0.00	2.53	0.01
Immigrant	-0.10	0.01	-9.60	0.00
Criminal record	-0.17	0.01	-22.73	0.00
Children * R: Left-right ideology	-0.01	0.00	-2.59	0.01

Table 13: Table of interaction results: Respondent left-right ideology vs. patient has criminal record

	Estimate	Std. Error	t value	Pr(>|t|)
(Intercept)	0.47	0.02	29.29	0.00
Chance of survival 50%	0.15	0.01	21.28	0.00
Chance of survival 80%	0.23	0.01	27.96	0.00
Male	-0.06	0.01	-8.49	0.00
44 years old	-0.00	0.01	-0.59	0.56
64 years old	-0.05	0.01	-7.63	0.00
Cook	-0.03	0.01	-5.14	0.00
Nurse	0.01	0.01	1.66	0.10
Professor	-0.06	0.01	-10.36	0.00
Has school children	0.21	0.01	22.81	0.00
Immigrant	-0.10	0.01	-9.52	0.00
Criminal record	-0.14	0.02	-5.89	0.00
R: Left-right ideology	0.00	0.00	1.47	0.14
Criminal record * R: Left-right ideology	-0.01	0.00	-1.48	0.14

Table 14: Table of interaction results: Respondent left-right ideology vs. patient immigrant status

	Estimate	Std. Error	t value	Pr(>|t|)
(Intercept)	0.38	0.02	24.79	0.00
Chance of survival 50%	0.15	0.01	21.56	0.00
Chance of survival 80%	0.23	0.01	27.48	0.00
Male	-0.06	0.01	-8.50	0.00
44 years old	-0.00	0.01	-0.59	0.56
64 years old	-0.05	0.01	-7.69	0.00
Cook	-0.03	0.01	-5.20	0.00
Nurse	0.01	0.01	1.75	0.08
Professor	-0.06	0.01	-10.17	0.00
Has school children	0.21	0.01	23.02	0.00
Immigrant	0.09	0.02	4.30	0.00
R: Left-right ideology	0.02	0.00	8.87	0.00
Criminal record	-0.17	0.01	-23.12	0.00
Immigrant * R: Left-right ideology	-0.03	0.00	-7.76	0.00

Table 15: Table of main results with distinction between EU and Non-EU immigrants

	Estimate	Std. Error	t value	Pr(>|t|)
(Intercept)	0.41	0.01	30.15	0.00
Chance of survival 50%	0.13	0.01	17.38	0.00
Chance of survival 80%	0.22	0.01	21.97	0.00
Male	-0.06	0.01	-5.46	0.00
44 years old	0.00	0.01	0.43	0.67
64 years old	-0.05	0.01	-6.61	0.00
Cook	-0.05	0.01	-8.84	0.00
Nurse	0.01	0.01	1.30	0.19
Professor	-0.05	0.01	-6.80	0.00
Has school children	0.20	0.02	13.09	0.00
German	0.06	0.01	10.96	0.00
Non-EU immigrant	0.01	0.01	0.81	0.42
Criminal record	-0.16	0.01	-17.04	0.00
